# GNSS Code Multipath Mitigation by Cascading Measurement Monitoring Techniques

**DOI:** 10.3390/s18061967

**Published:** 2018-06-19

**Authors:** Ali Pirsiavash, Ali Broumandan, Gérard Lachapelle, Kyle O’Keefe

**Affiliations:** Position, Location and Navigation (PLAN) Group, Schulich School of Engineering, University of Calgary, Calgary, AB T2N 1N4, Canada; abrouman@ucalgary.ca (A.B.); lachapel@ucalgary.ca (G.L.); kpgokeef@ucalgary.ca (K.O.)

**Keywords:** global navigation satellite systems (GNSS), multipath mitigation, measurement monitoring, multipath error estimation and measurement correction, stochastic model of measurements and weighting approach, multipath detection for excluding or de-weighing affected measurements

## Abstract

Various measurement monitoring techniques are investigated to mitigate the effect of global navigation satellite systems (GNSS) code multipath through error correction, stochastic weighting of measurements and detection and exclusion (or de-weighting) of affected measurements. Following a comprehensive review of each approach, the paper focuses on detection/exclusion and detection/de-weighting techniques where several single and dual-frequency monitoring metrics are employed in a combination with time-averaging and the *M of N* detection strategy. A new Geometry-Free (GF) detection metric is proposed given its capability to be combined with a preceding Code-Minus-Carrier (CMC)-based error correction to reduce the number of excluded or de-weighted measurements and thus preserve the measurement geometry. Three geometry-based algorithms, namely measurement subset testing, consecutive exclusion and iterative change of measurement weights are investigated to address multipath scenarios with multiple simultaneously affected measurements. Experimental results are provided using GPS L1, L2C and L5 data collected in multipath environments for static and kinematic scenarios. For GPS L1, the proposed combined method shows more than 38% improvement over a conventional Carrier-to-Noise-density ratio (C/N_0_)-based Least-Squares (LS) solution in all but deep urban canyons. Lower performance was observed for L2C and L5 frequencies with a limited number of satellites in view.

## 1. Introduction

Multipath is a major error source for global navigation satellite systems (GNSS) receivers. This phenomenon refers to the combination of Line-of-Sight (LOS) and a number of Non-Line-of-Sight (NLOS) components reflected off nearby obstacles one or more times before reaching the receiver antenna. Multipath signals differ from the LOS signal in power, code delay, carrier phase and frequency, all of which distort the correlation curve between the received signal and receiver-generated replica, resulting in pseudorange (code phase) errors of tens of metres [1]. Many multipath mitigation techniques have been developed in the literature, a review of which can be found in Reference [2]. Among these well-investigated multipath mitigation methods, receiver-level signal quality and integrity monitoring techniques have received much attention as they do not need major hardware modifications. A typical GNSS receiver consists of an RF front-end that down-converts and samples the RF signal, a signal processing stage where the signals are acquired, tracked and measurements are generated, and a navigation solution. Integrity monitoring can be performed at each of these stages. 

At the first stage, the output of the RF front end can be monitored, although this is challenging in multipath detection where spread GNSS signals are still buried under the noise floor [3]. At the navigation stage, Receiver Autonomous Integrity Monitoring (RAIM) is the most common technique. A thorough study of conventional and advanced RAIM algorithms is presented by Reference [4]. Conceptually, RAIM algorithms use measurement redundancy to identify outliers. Although effective for aviation applications, poor geometry and multiplicity of error sources make RAIM sub-optimal for land applications, particularly in urban areas with dense multipath [5]. Monitoring the quality of GNSS signals at the signal processing stage allows each Pseudorandom Noise (PRN) to be independently monitored, providing the capability of detecting multiple signal failures. Signal Quality Monitoring (SQM) has been well investigated for multipath detection and is usually implemented by adding additional monitoring correlators at the tracking stage. A comprehensive analysis of the sensitivity and effectiveness of SQM techniques has been reported in Reference [6]. While SQM techniques have their own limitations, especially in detecting short-range multipath, the other option is monitoring the GNSS measurements (i.e., code, carrier phase and Doppler). This is the main focus of this paper. In addition to code and carrier phase measurements, Carrier-to-Noise-density ratio (C/N_0_) is considered as a quality measurement for code multipath monitoring using the following three approaches.

1. Multipath Error estimation and measurement correction: The monitoring metrics defined in these techniques are generally based on combinations of erroneous (but unambiguous) code phase and precise (but ambiguous) carrier phase measurements to provide a direct measure of the code range multipath error, resulting in the possibility of pseudorange correction [1]. The Code-Minus-Carrier (CMC) metric is one of the well-known monitoring metrics used to characterize and measure code multipath errors by subtracting carrier phase measurements from corresponding pseudoranges (e.g., [7]). Since the pseudorange multipath error is considerably larger than that of the carrier phase, the code minus carrier measurement is mostly an indication of pseudorange multipath [8,9,10]. Although CMC can also be used in other multipath mitigation approaches such as multipath detection and exclusion [11], correction of affected measurements (when applicable) is preferred to mitigate the multipath error without geometry degradation. The major limitation is sensitivity to cycle slips due to signal attenuation, obstruction or antenna motion degrading the performance of the monitoring process [12,13,14].

2. Stochastic model of measurements and weighting approach: The second approach includes solutions where monitoring metrics are used in weighting schemes to mitigate the effect of stochastic errors. In this approach, the variances of the GNSS measurements are continuously adapted based on monitoring metrics where the contribution of degraded measurements are potentially de-weighted in the position solution. This requires defining a relationship between the metric outputs and degradation in measurement precision and sometimes tuning model parameters [15]. Besides the elevation angle of satellites [16], Signal-to-Noise Ratio (SNR) and equivalently C/N_0_ are well-known quality metrics investigated for the purpose of GNSS stochastic models and measurement weighting schemes. References [17,18,19] investigate the relationship between measurement precision and corresponding C/N_0_ to develop the *SIGMA-ɛ* and *SIGMA-Δ* stochastic models to cope with signal attenuation, diffraction and NLOS conditions. Although initially developed for carrier phase measurements, it has been shown that these models are also beneficial for pseudoranges as discussed by References [20,21,22]. More recently, Reference [23] exploited C/N_0_ measurements as a quality metric to weight GNSS measurements under multipath environments. Further investigations reported by Reference [24] examine the effectiveness of a C/N_0_-based weighting scheme for different multipath scenarios. Results show that although the C/N_0_-based solutions generally show improvement in positioning performance, as will be discussed later in this paper, they do not properly deal with gross errors caused by multipath. 

3. Multipath detection for excluding or de-weighing affected measurements: Modern multi-constellation receivers are equipped with hundreds of channels resulting in an unprecedented level of measurement redundancy. Checking for measurement failures and selecting those least distorted by gross errors such as multipath are much less likely to result in degraded geometry than in the past. This requires design and implementation of proper algorithms to detect and exclude (or de-weight) affected measurements. Note that the term “exclusion or de-weighting” is used here to emphasize the fact that beside excluding detected measurements, the effect of gross errors caused by multipath can be reduced through de-weighting but not fully excluding these measurements. Although similar in methodology, this concept differs from stochastic weighting of measurements (discussed above) where the variances of the GNSS measurements are continuously adapted based on designed monitoring metrics to mitigate the effect of stochastic errors. References [25,26] are a sample of recent work that show multipath errors can be specifically reduced by detecting and isolating the affected measurements when it does not significantly degrade solution geometry. 

Given the above, this paper investigates the use of measurement monitoring techniques to mitigate the effect of pseudorange multipath errors. In Section 2, a brief background is presented on the weighted Least-Squares (LS) solution for pseudorange-based positioning and the use of Dilution of Precision (DOP) to monitor the quality of the geometry. The three monitoring approaches, namely multipath error correction, stochastic weighting, and detection/exclusion (or-de-weighting) are discussed in Section 3. The main focus is on the third, where affected measurements are detected, excluded or de-weighted from the position solution. A C/N_0_-based multipath detection metric is investigated for a single-frequency receiver and two Differential C/N_0_ (DC/N_0_) and Geometric-free (GF)-based detection metrics are presented and compared for a dual (multi) frequency receiver. Detection, exclusion and de-weighting strategies, used in this research, are discussed in Section 4. Along with a combination of a time-averaging process and a *M of N* detection strategy, three geometry-based algorithms, namely measurement subset testing, consecutive exclusion, and iterative change of measurement weights, are evaluated. In Section 5, a combination of monitoring techniques is investigated based on two cascading methods and for more reliable multipath mitigation. Finally, the methods are tested in Section 6 using three sets of GPS L1, L2C and L5 measurements in static and kinematic multipath scenarios. 

In addition to analytical evaluation and practical implementation of the different monitoring techniques, the main contribution of this research includes: (a) Adaption of the GF metric to the case of multipath detection given its advantages over the conventional C/N_0_-based detection metrics, (b) investigation of new geometry-based exclusion and de-weighting approaches to address multiple simultaneous multipath scenarios and (c) cascading the different monitoring techniques to improve positioning performance. 

## 2. Background on Weighted Least-Squares Adjustment and Dilution of Precision (DOP)

In this section, a brief background is presented for weighted Least-Squares (LS) adjustment for a pseudorange-based positioning and the concept of weighted dilution of precision (DOP) as a quality metric for geometry monitoring. 

### 2.1. Weighted Least-Squares Adjustment for a Pseudorange-Based Positioning

The pseudorange-based position solution using least-squares adjustment is considered an optimal estimation of the state vector in the sense that it minimizes the sum of the weighted squared residuals. Since pseudorange is a nonlinear function of the receiver and satellite positions, to determine the receiver coordinates, the measurement model is linearized based on an initial estimate of receiver position and clock bias and the state vector is updated iteratively based on the estimated error vector as follows [1].

(1) $$\Delta \hat{x}={\left(H^{T}WH\right)}^{-1}H^{T}W\Delta P.$$

In Equation (1), for m pseudorange measurements, ΔP is the m×1 vector of differences between the updated and modeled pseudoranges based on the linearization point, often termed as the measurement misclosure vector. $\Delta \hat{x}$ represents the estimation of error in the state vector (or simply state error), including three unknown position elements plus receiver clock bias (in distance units), from the linearization point. **H** is the m×4 matrix of partial derivatives of the pseudoranges with respect to the unknowns and **W** denotes the weight matrix [1]. With this adjustment, the mean value for the estimated state error is determined as

(2) $$E\left\{\Delta \hat{x}\right\}=\Delta x+{\left(H^{T}WH\right)}^{-1}H^{T}WE\left\{e\right\},$$

where **e** is the m×1 vector of measurement errors and $E\left\{\Delta \hat{x}\right\}$ denotes the expected value of $\Delta \hat{x}$. Equation (2) means that some portion of any systematic errors or biases in the measurements will be absorbed by state estimation depending on weight matrix and receiver-satellite geometry [27]. The covariance matrix for the state error, called precision matrix, is also determined as

(3) $$C_{\Delta \hat{x}}=E\left\{\left(\Delta \hat{x}-E\left\{\Delta \hat{x}\right\}\right){\left(\Delta \hat{x}-E\left\{\Delta \hat{x}\right\}\right)}^{T}\right\}={\left(H^{T}WH\right)}^{-1}\left(H^{T}WC_{e}WH\right){\left(H^{T}WH\right)}^{-1}$$

where $C_{e}$ is the covariance matrix of measurement errors expressed as

(4) $$C_{e}=E\left\{\left(e-E\left\{e\right\}\right){\left(e-E\left\{e\right\}\right)}^{T}\right\}=\sigma_{0}^{2}Q_{R},$$

where $\sigma_{0}^{2}$ is the a priori variance factor [1,8]. $Q_{R}$ is the cofactor matrix of the measurements. For an uncorrelated set of measurements, $Q_{R}$ is a diagonal matrix whose diagonal elements multiplied by a priori variance factor correspond to measurements variances ($\sigma_{i}^{2}\;\mathrm{for}\;i=1,\;2,\;\ldots ,\;n$). On the right-hand side of Equation (3), the product of the symmetric and positive definite matrices will increase the diagonal elements of the resulting matrix. Therefore, to reduce their products and heuristically minimize $C_{\Delta \hat{x}}$, a convenient method is to set $W=C_{e}{}^{-1}$.

(5) $$\begin{array}{cc} C_{\Delta \hat{x}} & ={\left(H^{T}C_{e}{}^{-1}H\right)}^{-1}\left(H^{T}C_{e}{}^{-1}C_{e}C_{e}{}^{-1}H\right){\left(H^{T}C_{e}{}^{-1}H\right)}^{-1}\\ & ={\left(H^{T}C_{e}{}^{-1}H\right)}^{-1}\left(H^{T}C_{e}{}^{-1}H\right){\left(H^{T}C_{e}{}^{-1}H\right)}^{-1}\\ & ={\left(H^{T}C_{e}{}^{-1}H\right)}^{-1}=\sigma_{0}^{2}Q_{P}, \end{array}$$

where $Q_{P}$ is the cofactor matrix of the state error estimation. Equations (3) and (5) mean that to minimize the effect of stochastic errors, the measurements should be weighted based on a proper estimate of their variance values. This concept will be used in Section 3.2 to develop a stochastic model of measurements to mitigate the effect of stochastic errors caused by multipath. When multipath is detected as a gross error, its effect can be mitigated by decreasing the contribution of the detected measurements in the position solution. This can be done for example by deliberately de-weighting measurements as will be discussed in Section 4.2. Although similar in methodology, these two approaches differ as the first one deals with the stochastic effect of multipath (for example in high dynamic scenarios where multipath behaves more like a noise than bias) while the second deals with multipath gross errors.

### 2.2. Measurement Geometry and DOP

In Equation (5), $Q_{P}$ is weighted by the cofactor matrix of measurements ($Q_{R}$) and is a function of **H**, which itself is a function of the receiver-satellite geometry. Because the diagonal elements of $Q_{P}$ are typically greater than one, the precision is diluted. Therefore, these diagonal elements are used to constitute quality metrics for measurement geometry called (weighted) Dilution of Precision (DOP) [1]. Assuming the position errors are estimated in east, north and vertical (up) local coordinate frame, the corresponding East DOP (EDOP), North DOP (NDOP) and Vertical DOP (VDOP) values can be computed based on the square root of the diagonal elements of $Q_{P}$ [1]:

(6a) $$\mathrm{EDOP}=\sqrt{{\left(Q_{P}\right)}_{\Delta E}},$$

(6b) $$\mathrm{NDOP}=\sqrt{{\left(Q_{P}\right)}_{\Delta N}},$$

(6c) $$\mathrm{VDOP}=\sqrt{{\left(Q_{P}\right)}_{\Delta V}}.$$

The same approach is also considered for time estimation and corresponding Time DOP (TDOP) value. The Position DOP (PDOP) which relates to the three-dimensional (3D) position solution, is also computed as

(7) $$\mathrm{PDOP}=\sqrt{{\left(\mathrm{EDOP}\right)}^{2}+{\left(\mathrm{NDOP}\right)}^{2}+{\left(\mathrm{VDOP}\right)}^{2}}.$$

The same methodology can be applied for geometry DOP which also involves TDOP values in its definition. The PDOP values presented here will be used in Section 4.2 to develop the proposed geometry-based exclusion and de-weighting algorithms. 

## 3. Measurement Monitoring Techniques for Multipath Mitigation

In this section, the CMC-based multipath error correction, C/N_0_-based stochastic weighting and detection of affected measurements are discussed in details. While the two first approaches have been previously investigated in the literature [8,9,10,11,12,13,14,15,16,17,18,19,20,21,22,23,24], the focus will be on detection techniques where a new Geometric-Free (GF)-based multipath detection is presented. The detection results are then used in the following sections to exclude or de-weight the affected measurements. 

### 3.1. Multipath Error Estimation and Measurement Correction

This approach is generally based on a direct measure of code range multipath error, thereby offering the possibility of pseudorange correction. As discussed before, CMC is one of the well-known monitoring metrics commonly used in the literature as a basic measure of code multipath error estimation and correction.

- Code-Minus-Carrier (CMC) for multipath estimation and correction

CMC is computed by subtracting the carrier phase measurements from the corresponding pseudoranges to remove the effect of non-dispersive systematic errors such as receiver and satellite clock errors, orbital errors and tropospheric delays. By doing so, the CMC metric for the *i*th satellite at *k*th measurement epoch is calculated as [8,9,10]:

(8) $$\begin{array}{cc} CMC_{i}^{}\left(k\right) & =P_{i}^{}\left(k\right)-\phi_{i}^{}\left(k\right)\\ & =MP_{P,i}^{}\left(k\right)+\left(2d_{ion,i}\left(k\right)-\lambda N_{i}^{}\left(k\right)\right)+\left(\eta_{P,i}^{}\left(k\right)-MP_{\phi ,i}^{}\left(k\right)-\eta_{\phi ,i}^{}\left(k\right)\right), \end{array}$$

where $P_{i}^{}\left(k\right)$ and $\phi_{i}^{}\left(k\right)$ are the corresponding code and carrier phase measurements (both converted to units of length). $d_{ion,i}\left(k\right)$ is ionospheric delay, λ and $N_{i}^{}\left(k\right)$ are wavelength and ambiguity, and $MP_{P,i}^{}\left(k\right)$, $\eta_{P,i}^{}\left(k\right)$, $MP_{\phi ,i}^{}\left(k\right)$, $\eta_{\phi ,i}^{}\left(k\right)$ are code and carrier multipath and noise errors respectively. When a carrier-phase measurement is not available, an accumulated delta range based on Doppler measurements can be used although it will be noisier [28]. It is shown that in addition to a code multipath error, the result includes carrier phase ambiguities and twice the ionospheric errors (due to ionospheric code delay and phase advance) plus (code and carrier) noise and carrier phase multipath. Considering that the integer ambiguity is constant during a cycle slip-free period and ionosphere changes are low frequency, their effects can be removed based on a simple or cumulative moving average (see Appendix A) sufficiently short to avoid biases due to changing geometry. Therefore, the pseudorange multipath error can be extracted by a CMC-based monitoring metric as

(9) $$m_{cmc,i}^{}\left(k\right)=CMC_{i}^{}\left(k\right)-{\langle CMC_{i}^{}\rangle }_{k},$$

where ${\langle CMC_{i}^{}\rangle }_{k}$ denotes the mean value of the CMC metric at the *k*th epoch computed by a moving average (see Appendix A). The output of the CMC metric is a direct measure of the code multipath error which can be directly used for pseudorange correction without geometry degradation, which is the case when excluding or de-weighting measurements:

(10) $${\hat{P}}_{i}^{}\left(k\right)=P_{i}^{}\left(k\right)-m_{cmc,i}^{}\left(k\right),$$

where ${\hat{P}}_{i}^{}\left(k\right)$ is the corrected pseudorange. Assuming a cumulative moving average, Equation (10) is another form of carrier-smoothing of code pseudoranges (proof in Appendix B). Due to the dependency of the CMC on carrier phase measurements, the major limitation is the need to restart the time averaging process in the event of a cycle slip. Using pre-defined modeling or a reference receiver to remove the mean value is not possible since the effect of integer ambiguity differs from receiver to receiver. 

### 3.2. Stochastic Model of Measurements and Weighting Approach

Besides the constant and elevation-based weighting models, C/N_0_-based models have been widely investigated in the literature for the purpose of measurement weighting. As shown by References [20,21,22], a lower C/N_0_ results in less precise range measurements. Therefore, according to the discussion conducted in Section 2.1, the measured C/N_0_ can be used as a quality metric to model measurement weights in position solutions.

- Carrier-to-Noise-density ratio (C/N_0_) metric for stochastic modeling

Reference [17] presents a relationship between measurement precision and the corresponding C/N_0_ which was then used to develop a stochastic model for the GNSS code and carrier phase measurements [19,20,21,22]. The variance of a measurement from the *i*th satellite at the *k*th measurement epoch is then defined as

(11) $$\sigma_{i}^{2}(k)=a+b\times 10^{\frac{-C/N_{0}\left(k\right)}{10}},$$

where *a* (in m^2^) and *b* (in m^2^Hz) are model parameters and are set based on multipath environment and user equipment [20,21,22,23]. $C/N_{0}\left(k\right)$ is the measured ratio of the received carrier power to the noise density (either smoothed or not), in dB-Hz and at the *k*th measurement epoch. This model (sometimes called the *SIGMA-ɛ* model) is based on the assumption that measurements with C/N_0_ value generally have higher precision which can be also effective in mitigating the stochastic errors caused by multipath. In addition to using the absolute value of the C/N_0_ measurements, a *SIGMA-Δ* model was also developed by Reference [18] to use measured C/N_0_ value differences and a corresponding pre-defined template to de-weight distorted measurements. Such a template can be obtained with either data recording with a specific antenna, use of an undisturbed reference receiver or simply by the use of a sliding moving average window to determine the nominal mean of C/N_0_ measurements. Each method has its own limitations and challenges and, even if the nominal value can be estimated, relating the multipath-caused deviation to the precision of the measurements is still unknown and requires model tuning. 

### 3.3. Multipath Detection for Excluding or De-Weighing Affected Measurements

Along with the stochastic modeling of the measurements, gross errors caused by multipath can be specifically reduced by detecting and excluding or de-weighting affected measurements. Thanks to the development of multi-GNSS constellation receivers, this approach is increasingly effective as the redundancy is sufficiently large to detect and exclude (or de-weight) faulty measurements. Two conventional C/N_0_-based detection metrics are discussed and a new Geometry-free (GF) monitoring metric is then presented for more reliable multipath detection.

- C/N_0_ as a single-frequency monitoring metric for detection

In addition to being used in stochastic modelling of measurements, C/N_0_ is conventionally used for detection of degraded measurements [29,30]. Under multipath conditions, C/N_0_ fluctuates as a result of the superposition of direct and one or more reflected signals. These fluctuations can be monitored to de-weight or exclude affected measurements.

- Differential C/N_0_ (DC/N_0_)-based monitoring metric for detection

Having measurements on more than one carrier frequency provides auxiliary information that can be used for multipath detection. Depending on the phase lag of the reflected signal with respect to the direct signal, multipath interference on the measured C/N_0_ can be either constructive or destructive. However, as the phase lag is frequency dependent, the C/N_0_ is affected differently on each frequency. Thus, by differencing C/N_0_ measurements, multipath can be detected. The desired detection metric is defined as

(12) $$m_{DC/N_{0},i}\left(k\right)={\left(C/N_{0}\right)}_{i}^{f1}\left(k\right)-{\left(C/N_{0}\right)}_{i}^{f2}\left(k\right)-\Delta C_{i}^{f1f2}\left(k\right),$$

where $m_{DC/N_{0},i}\left(k\right)$ is the differential C/N_0_-based detection metric for the *i*th satellite at the *k*th monitoring epoch, ${\left(C/N_{0}\right)}_{i}^{f1}(k)$ and ${\left(C/N_{0}\right)}_{i}^{f2}(k)$ the corresponding C/N_0_ values (in dB-Hz) for *f*1 and *f*2 frequencies and $\Delta C_{i}^{f1f2}\left(k\right)$ is the corresponding nominal C/N_0_ differences (between *f*1 and *f*2) under multipath-free conditions. $\Delta C_{i}^{f1f2}\left(k\right)$ is mostly a function of satellite elevation due to non-proportional frequency-based receiver antenna gain for different elevation angles. $\Delta C_{i}^{f1f2}\left(k\right)$ can either be estimated by time-averaging or modeled through calibration or the use of a reference antenna. As an advantage, differencing two or more C/N_0_ measurements (in dB-Hz) from different carrier frequencies cancels out the effect of frequency-independent and proportional frequency-dependent parameters and the rest can be calibrated more easily based on receiver antenna gain for different arrival directions. By differencing the measured C/N_0_ values on two frequencies, two scenarios are considered. First, when the deviation from the nominal value occurs with opposite signs on two frequencies the multipath signature is magnified. Second is the case when the C/N_0_ deviations occur with the same sign on two frequencies resulting in lower multipath visibility in the output of the differential metric. Hence, the detection procedure will be more reliable if three (or more) frequencies are used since the phase of the multipath on three frequencies is less likely to be consistent than two [30]. The output of C/N_0_ differences will also be an indication of multipath for at least one of the constituent frequencies, but it is not possible to distinguish which one. For both single-frequency C/N_0_ and DC/N_0_-based detection metrics, for a given Signal-to-Multipath Ratio (SMR), a shorter multipath delay imposes a greater effect on the monitoring metrics because the direct and reflected correlation peaks will be closer to each other, resulting in a larger distortion of the computed C/N_0_ value. This means that the C/N_0_-based detection metrics will be more sensitive to short-delay multipath, which imposes smaller errors on pseudoranges compared, for example, to a medium-delay multipath. In this regard, to cover a wider range of multipath scenarios, the C/N_0_-based detection metrics should be used along with other monitoring techniques that are more sensitive to medium and long-delay multipath (e.g., signal quality monitoring metrics defined at the receiver tracking stage [6,24]). Moreover, for a given signal modulation and reflection path delay, the C/N_0_ value will be more affected for lower chipping-rate signals such as GPS L1 C/A and Galileo E1 than for high chipping-rate ones, such as GPS L5 and Galileo E5 [29].

- Geometry-free (GF) monitoring metric for detection

Multipath can also be detected through the difference between pseudoranges on two frequencies. This combination, denoted $m_{GF,i}^{f1f2}\left(k\right)$, cancels the geometric part of the measurements, leaving the frequency-dependent effects (mainly ionospheric delay) besides multipath and measurement noise:

(13) $$m_{GF,i}^{f1f2}\left(k\right)=P_{i}^{f1}\left(k\right)-P_{i}^{f2}\left(k\right)-\Delta d_{ion}^{f1f2}\left(k\right),$$

where $\Delta d_{ion}^{f1f2}\left(k\right)$ is the difference in the ionospheric effects and can be estimated and removed either through modeling the use of a nearby reference receiver or time-averaging; the remainder can be used to monitor the code multipath. Similar to the DC/N_0_-based monitoring metric, the GF metric is a combination of errors on two frequencies and thus does not provide separate information about multipath on each frequency. It can thus be used only for satellite-by-satellite detection and exclusion (or de-weighting). The GF monitoring metric is directly proportional to code multipath errors and from this point of view outperforms C/N_0_-based detection metrics (including both single-frequency C/N_0_ and DC/N_0_-based detection metrics) which are more sensitive to short-delay multipath with a small impact on code tracking errors. The main advantage of the GF metric is its capability to be combined after a CMC-based error correction. Multipath errors can be first reduced by applying CMC-based error corrections and then the GF detection metric can be formed by differencing corrected pseudoranges on two frequencies. This will be discussed in Section 5 to perform a complementary combination of the monitoring techniques. 

## 4. Detection, Exclusion and De-Weighting Strategies

Specific detection, exclusion and de-weighting strategies are now discussed. Besides a customized implementation of time-averaging and a *M of N* detection strategy, three new geometry-based exclusion algorithms are investigated. 

### 4.1. Detection Strategy

The detection process can be considered as a maximization of a likelihood ratio by setting an appropriate detection threshold for each PRN. To determine the threshold, the nominal multipath-free characteristics of the detection metrics must be investigated first and an appropriate probability of false alarm must be selected [20,21,31]. Since either calibration, pre-defined models or reference receivers cannot be used to remove the mean value of the CMC metric (see Section 3.1), a moving average is applied. The performance will be limited by two factors. First, as the code-multipath error is not zero-mean [32], low-frequency biases caused by multipath will be filtered out, which is not desirable for the multipath detection purpose. Second, the performance of time averaging will be limited by the sliding window length. The larger length provides larger sample-space and thus results in more precise estimation of the mean value at the expense of a delayed response to multipath transition states, geometry changes and other signal degradations such as signal attenuation and blockage. To balance these two limitations, the moving average window is set to at least one multipath period, which in static cases may reach several minutes. The nominal Standard Deviation (SD) of the monitoring metrics in the absence of multipath was determined using reference data collected in a low multipath environment. 

The detection procedure is then defined based on a fixed interval detector called the *M of N* detection strategy. The *M of N* detector is a fixed-lag sliding window which takes a window of *N* samples (based on current and *N −* 1 preceding samples) and compares them to a predefined threshold. If *M* or more samples exceed the threshold, then the detection output will be 1 and otherwise 0. This procedure is then repeated for the next window in the search pattern. With this detection strategy, the overall probability of false alarm in *N* trials is given by [1]

(14) $$P_{FA}=\sum_{n=M}^{N}\left(\begin{array}{c} N\\ n \end{array}\right)P_{fa}^{n}{\left(1-P_{fa}^{}\right)}^{N-n}=1-\sum_{n=0}^{M-1}\left(\begin{array}{c} N\\ n \end{array}\right)P_{fa}^{n}{\left(1-P_{fa}^{}\right)}^{N-n},$$

where $\left(\begin{array}{c} N\\ n \end{array}\right)$ is the number of combinations of N items taken n at a time. $P_{fa}^{}$ is the false alarm probability in each trial and equals 0.27% under a normal distribution assumption and a ±3SD detection threshold. In the case of (*N*, *M*) = (1, 1), the detection strategy is considered as a general likelihood ratio test by comparing each sample with the detection threshold at each epoch. A proper selection of *N* and *M* can decrease the false alarm probability while imposing latency in the transition from the null (when there is no or low multipath) to the alternate (when multipath occurs) hypothesis and vice versa [1,33]. Given the periodic nature of GNSS multipath (or even when multipath behaves more like noise in high dynamic scenarios), a side effect of the latency will be a reduction in the rate of change between the two detection states. In the case of exclusion, the lower rate of change has the benefit of smoothing the position results. In Section 6, this strategy will be applied to samples of the monitoring metrics to detect the effect of multipath. For each scenario, the appropriate parameters will be set based on the desired false alarm probability and multipath conditions.

### 4.2. Exclusion and De-Weighting of Affected Measurements

Once multipath is detected, its effect can be reduced by excluding or de-weighting the affected measurement(s). The critical point is the effect of exclusion or de-weighting on measurement geometry. Poor geometry may magnify the effect of remaining errors on position solution. Since position is the major concern, PDOP is adopted as a quality metric to monitor the effect of exclusion or de-weighting on measurement geometry. The strategy allows the exclusion or de-weighting of detected measurements for the epochs where the corresponding PDOP values, before and after exclusion or de-weighting, remain at a fair level (e.g., below 8 as discussed by [20,34]). The following three geometry-based approaches are investigated. Although not based on rigorous statistical theory, the proposed methods can reduce or potentially eliminate the effect of multipath, as it will be shown in the data analysis section. 

**Measurement subset testing:** This includes exclusion of a subset of the detected measurements which have the least impact on measurement geometry below the tolerable PDOP threshold. This is done by calculating PDOP for several different combinations of detected measurements in order to locate the subset with the least impact on measurement geometry. Although optimized in finding the subset with least impact on geometry, measurement subset testing is computationally heavy. With $N_{s}$ satellites tracked and $N_{d}$ detected as affected by multipath, the $\sum_{n=1}^{N_{d}}\left(\begin{array}{c} N_{s}\\ n \end{array}\right)$ combinations must be tested. Due to the computational burden of this method, an alternative solution is considered in this research labeled *consecutive exclusion* and is explained as follows.

**Figure 1:**
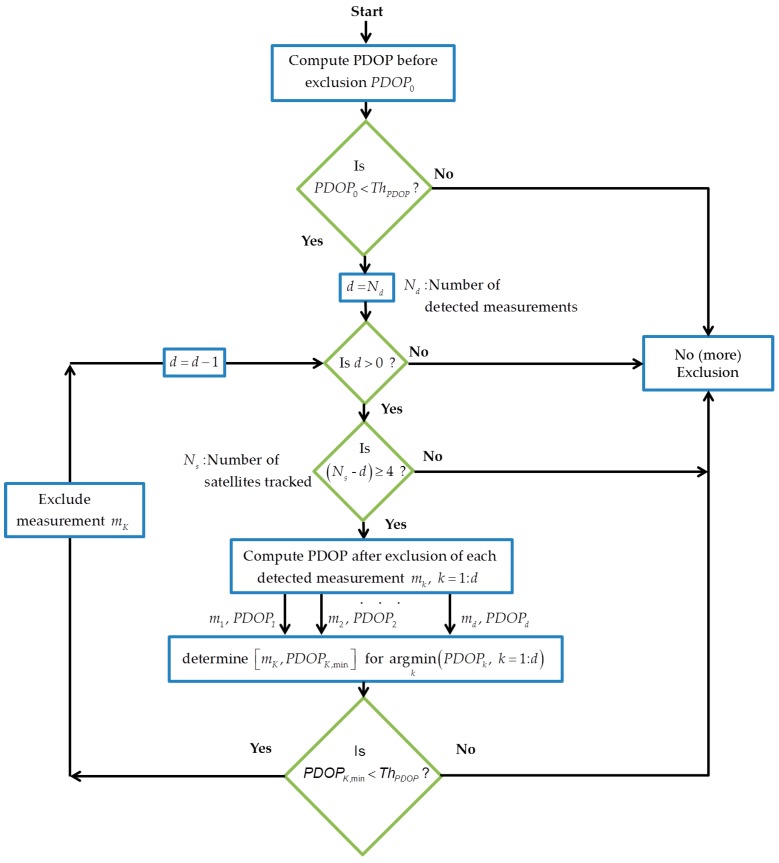
Consecutive Exclusion: Based on applying a series of consecutive tests to exclude the detected measurements until the tolerable PDOP threshold is satisfied. Exclusion priority is defined based on the effect of each single exclusion on measurement geometry.

**Consecutive exclusion:** Detected measurements can be consecutively excluded until the PDOP exceeds a tolerable threshold. The detected measurements are sorted based on an exclusion priority, which can be determined based on different criteria such as the value of corresponding CMC, C/N_0_, elevation and residuals or the effect of each on the measurement geometry. The last criterion is used in this paper where the maximum number of PDOP adjustments is given by $\sum_{i=0}^{N_{d}-1}\left(N_{d}-i\right)$. Figure 1 shows this procedure when the effect of each exclusion on measurement geometry is considered as the exclusion priority. Although not optimized to determine the best subset in terms of geometry, consecutive exclusion is preferred to subset testing when the number of affected measurements increases due to its lower computational burden. In the case of a single multipath detection, both methods are equivalent [35]. This methodology is similar to the *Data Snooping* reliability test that is usually performed using least-squares residuals [27].

**Figure 2:**
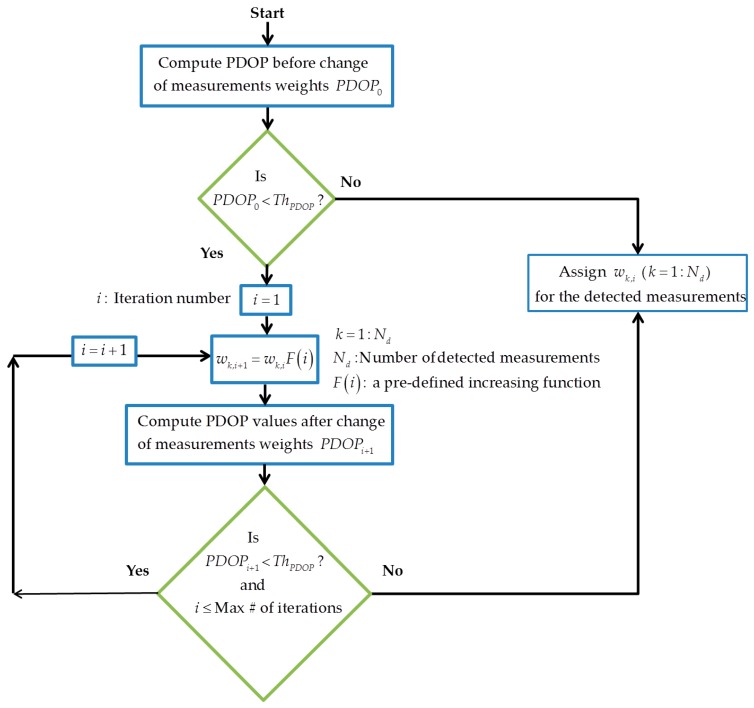
Iterative change of measurement weights (measurement de-weighting) below the tolerable PDOP threshold and limited number of iterations.

**Iterative change of measurement weights or de-weighting**: This method retains all measurements but iteratively reduces their contribution to the solution if multipath is detected. This differs from measurement stochastic weighting which aims to deal with stochastic errors rather than gross errors caused by multipath. The main idea is based on the *Danish method* [20,21] which changes the weight of measurement according to normalized residuals. Here, based on the monitoring results, the procedure iteratively decreases the contribution of the detected measurements by increasing the corresponding variance factors. The method continues until the tolerable PDOP threshold is exceeded. Figure 2 shows the geometry-based iterative change of measurement weights using a pre-defined increasing function (e.g., linear or exponential). Although, this procedure does not absolutely eliminate multipath errors, when the number of measurements is limited and exclusion is not desirable, de-weighting of affected measurements can be useful. The computational burden depends on the increasing function and the maximum number of iterations required to reach the predefined geometry threshold. 

## 5. Combination of Monitoring Techniques for a Reliable Multipath Mitigation

None of the techniques presented in Section 3 are completely reliable and each has its own limitations in different multipath situations. Therefore, a complementary combination of the aforementioned techniques is investigated for more reliable multipath mitigation. This is done here in two cascading approaches as follows.

**Figure 3:**
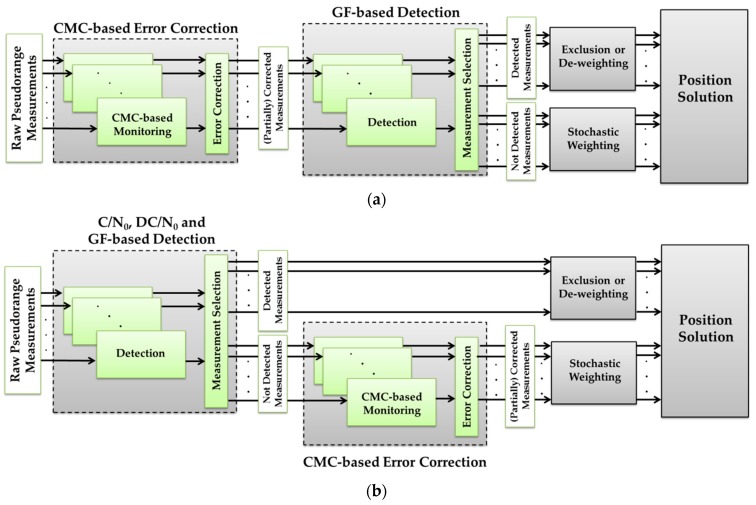
Combination of monitoring techniques in two cascading approaches, (**a**) a preceding CMC-based error correction followed by a GF-based multipath detection and exclusion (or de-weighting), and stochastic weighting, (**b**) a preceding multipath detection (for exclusion or de-weighting) followed by a CMC-based error correction and stochastic weighting.

First CMC-based error corrections are applied to pseudoranges to alleviate multipath errors and then the GF-based detection metrics are used to exclude (or de-weight) remaining errors below a tolerable PDOP threshold. For example, consider a scenario when GPS signals are available on L1, L2C and L5. At each epoch of GPS L1 positioning (when only L1 pseudoranges are used in the position solution, but measurements on other frequencies are available for monitoring purposes), first CMC-based error correction is applied on all three frequencies. The GF detection metrics are then computed using L1-L2C and L1-L5 combinations with the (partially) corrected measurements. Multipath is detected if at least one of the corresponding *M of N* detection outputs is one. It is noted that the C/N_0_-based detection metrics cannot be used to detect multipath on previously (but partially) corrected measurements. Detection results are then exploited in exclusion or iterative de-weighting approaches depending on the multipath environment and measurement geometry (see Section 4.2). For included measurements or for those not de-weighted iteratively, an elevation or C/N_0_-based model is applied to mitigate the effect of stochastic errors. Figure 3a shows the flowchart of this procedure. In this approach, the preceding CMC-based error correction potentially reduces the number of measurements excluded or de-weighted in position solutions and thus reduces measurement geometry degradation. However, since all GPS satellites do not broadcast signals on all three frequencies at this time, the GF-based detection/exclusion (or de-weighting) performance is limited to the PRNs with signals on at least two different frequencies. For example, for GPS L2C or L5 positioning (when GPS signals are monitored on L1, L2C and L5 frequencies, but only L2C or L5 pseudoranges are respectively used for positioning), at least one of the GF-based detection metrics is configurable by involving the corresponding L1 measurements. For GPS L1 positioning, the multipath errors (not corrected by CMC) without corresponding L2C or L5 measurements will remain undetected.

The second approach is first exclusion (or de-weighting) of detected measurements (based on the union of all detection metrics) and then correction of multipath errors on included (or not de-weighted) PRNs by applying CMC-based error correction. The flowchart of this procedure has been illustrated in Figure 3b. In this approach, multipath is first detected based on the union of detection results extracted by all related C/N_0_, DC/N_0_ and GF-based detection metrics. For example, for GPS L1 positioning, the combination of detection metrics will include the single-frequency C/N_0_-based detection metric on L1 frequency, the DC/N_0_ and GF-based detection metrics on L1-L2C and L1-L5 differences. At each epoch, multipath is detected if at least one of the corresponding *M of N* detection outputs is one. The detection results are then used to exclude or de-weight the affected ones. The CMC-based correction is applied on included (or not de-weighted) measurements followed by an elevation of C/N_0_-based stochastic models. In this approach, since the single-frequency C/N_0_-based detection is involved, besides the differential metrics, there is at least one detection metric configurable for each PRN. The drawback is the possibility of excluding (or de-weighting) measurements whose errors can be potentially corrected by a preceding CMC-based error correction.

In comparison, as three and four GNSS frequencies are becoming more common, the first approach is preferred to reduce the number of exclusions and thus preserve measurement geometry. However, with the current GNSS constellation, where a significant number of satellites transmit on only two frequencies, or for users that can only receive one or two frequencies, the second approach offers higher detection performance. This is because the second combined method exploits single-frequency C/N_0_-based detection besides the other differential metrics. As mentioned previously, the drawback is the possibility of excluding (or de-weighting) measurements whose errors can be potentially corrected by a preceding CMC-based error correction. In the following data analysis section, for different test scenarios, both combined methods will be investigated in advance, and one of them will be chosen based on the test scenario and multipath conditions. 

## 6. Field Data Analysis

GPS L1 C/A, L2C (M + L) and L5 (I + Q) code, carrier and C/N_0_ observables were collected (every one second) in multipath environments to evaluate the performance of the monitoring techniques discussed before. The ephemeris data was also collected to extract satellite positions and clock corrections required for position solutions. First, the CMC-based monitoring metrics and C/N_0_ measurements are monitored for multipath error correction and a stochastic weighting model, respectively. For detection, exclusion and de-weighting purposes, the performance of the C/N_0_, DC/N_0_ and GF-based detection metrics are first evaluated and detection results are then used to exclude or de-weight the measurements affected. Finally, the combination of the CMC-based error correction and detection/exclusion or detection/de-weighting techniques are investigated in a C/N_0_-based stochastic model. 

### 6.1. Static Test

**Figure 4:**
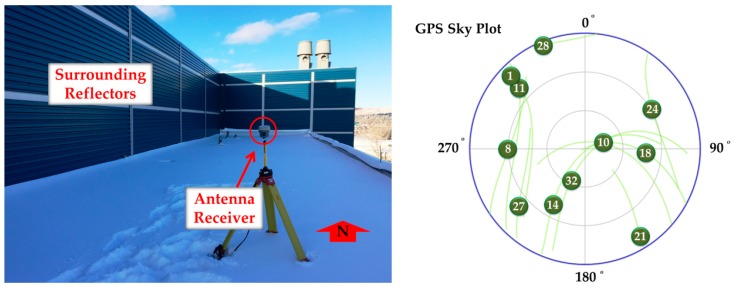
Multipath data collection using a Trimble R10 antenna-receiver surrounded by reflectors—Static test scenario.

A Trimble R10 GNSS receiver (Trimble Inc., Sunnyvale, CA, USA) was used to collect GPS L1, L2C and L5 measurements. Figure 4 shows the equipment, its location on a rooftop near reflective surfaces, and the available satellite geometry during the test. The antenna location was pre-surveyed and tied to the International Terrestrial Reference Frame 2013 (ITRF) to derive position error results in Section 6.1.2. 

#### 6.1.1. Monitoring Results

**Figure 5:**
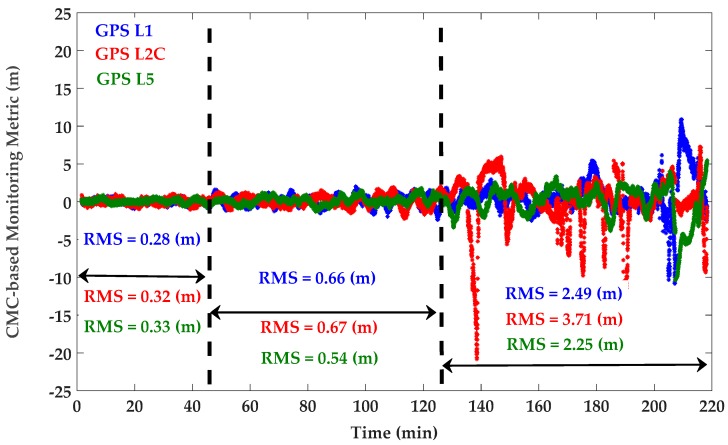
CMC-based monitoring metric for GPS L1 C/A (blue), L2C M + L (red), L5 I + Q (green)—PRN 10 affected by different multipath levels.

**Figure 6:**
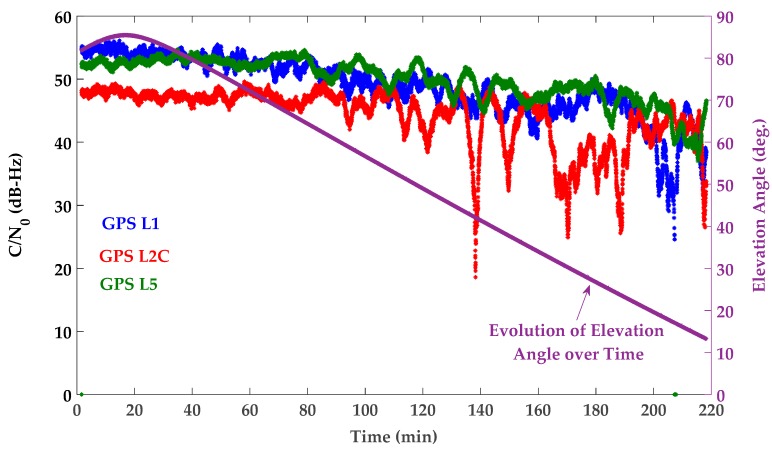
Left vertical axis: C/N_0_ measurements for PRN 10—GPS L1 C/A (blue), L2C M + L (red), L5 I + Q (green)—Right vertical axis: Evolution of elevation angle over time for PRN 10 (purple).

The time-averaging approach (discussed in Section 4.1) was used to estimate the mean value of the different monitoring metrics. A simple moving average was used with the length of 10 min to take into account the effect of static multipath oscillations. The 10 min interval was selected based on twice the average period observed for the quasi-periodic oscillations of the all PRNs exhibiting static multipath [32]. While similar results were observed for all PRNs, PRN 10, affected by different levels of multipath, is used to examine the sensitivity of the detection procedure. At each epoch, the CMC metric was obtained by subtracting carrier phase measurements from corresponding pseudoranges according to Equation (8). The nominal mean value was then filtered out to extract the CMC-based monitoring metric defined in Equation (9). This was done using the moving average discussed above. Since the cycle slips may result in a new unknown ambiguity in the carrier phase measurements [1,8], the moving average buffer was reset if a cycle slip was detected. The cycle slip detection procedure was performed based on the phase velocity trend method with a threshold of 1 cycle [36]. Since the pseudorange multipath error is considerably larger than that of the carrier phase, the CMC-based monitoring metric is mostly an indication of code (pseudorange) multipath as discussed in Section 3.1. For PRN 10, Figure 5 shows the L1 C/A, L2C and L5 CMC-based monitoring metrics as an indication of the corresponding code multipath errors. The corresponding Root-Mean-Square (RMS) values have also been presented where measurements are divided in three levels in terms of multipath error. As seen in Figure 4, PRN 10 travels on the southeast side of the antenna from high to low elevation and surrounding reflectors on the west side create multipath. Generally, higher multipath was observed on L2C as expected due to the relatively lower chipping rate of the latter [0.5115 MHz for L2C (M and L) compared to 1.023 MHz for L1 C/A and 10.23 MHz for L5 (I and Q)] and signaling bandwidth [20.46 MHz for L2C (M and L) compared to 24 MHz for L5 (I and Q)] [37,38]. Besides multipath characterization, the extracted CMC-based monitoring metric is used in the following subsections to correct the corresponding pseudoranges according to Equation (10). In addition to code and carrier phase measurements (and thus CMC metric), multipath affects the measured signal power and C/N_0_ values as shown in Figure 6. The carrier and noise power and thus C/N_0_ are estimated by the receiver, based on in-phase and out-of-phase correlator outputs [39]. Affected by the time-varying phase lag between the direct and reflected signal(s), as they add constructively or destructively with each other, the power of the combined signal and consequently the measured C/N_0_ fluctuate with time. Since the phase lag is frequency dependent, the C/N_0_ is affected differently on each frequency [32]. Figure 6 shows the corresponding C/N_0_ measurements where the constructive and destructive effects of multipath are observed on each frequency with different phase lags. For each PRN, the measured C/N_0_ will be used (in the next subsections) to perform the stochastic weight scheme defined by Equation (11).

**Figure 7:**
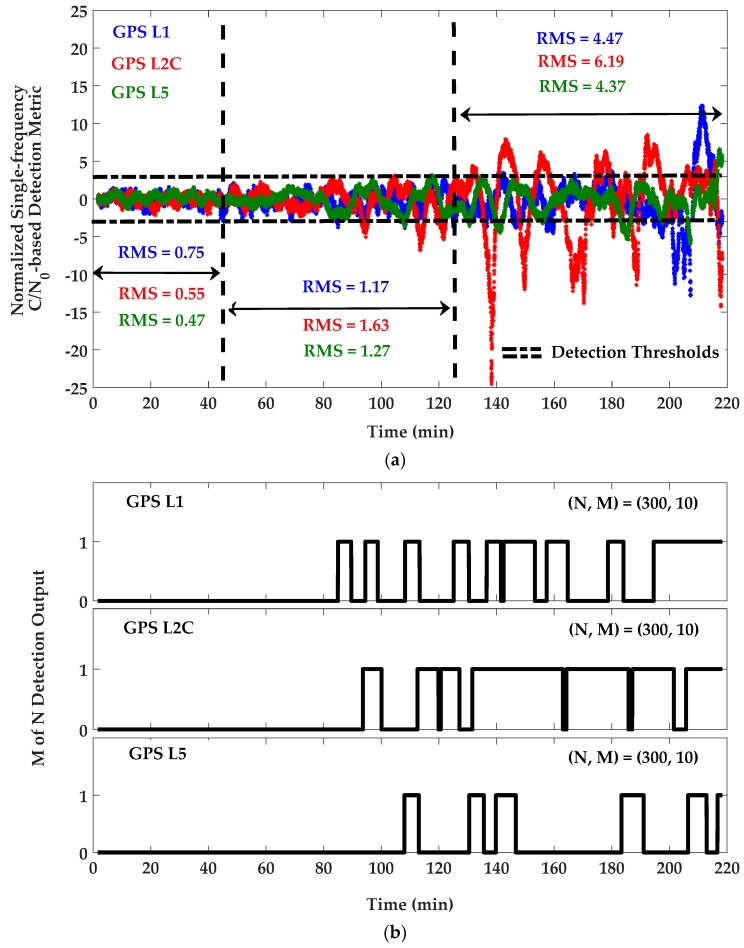
(**a**) Single-frequency C/N_0_-based detection metric and (**b**) *M of N* detection outputs for GPS L1/L2C and L5 frequencies—PRN 10.

**Figure 8:**
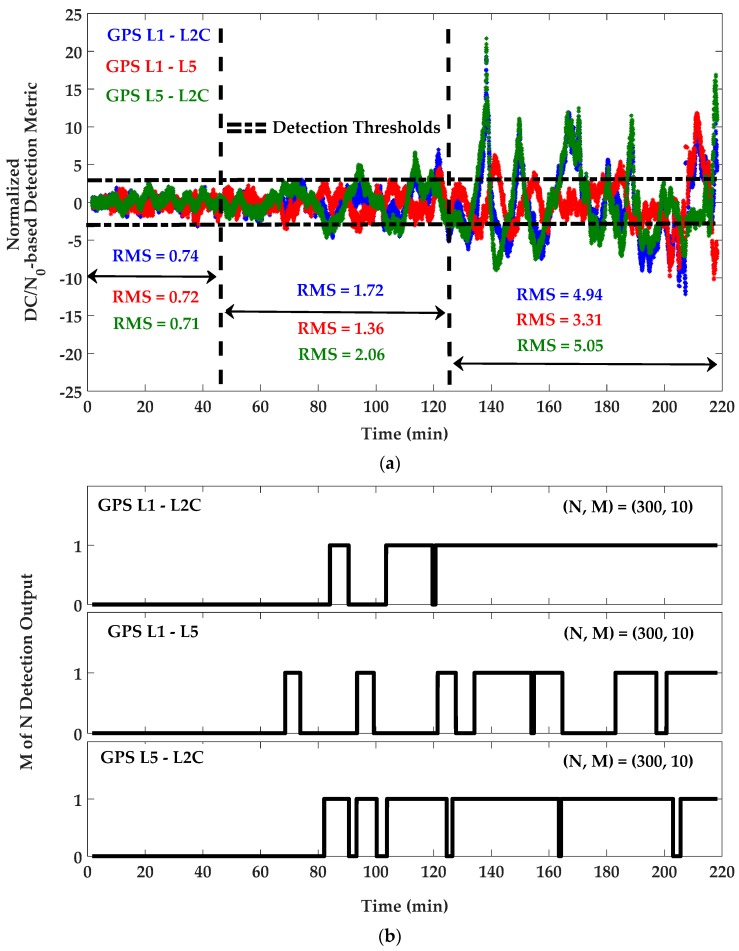
(**a**) DC/N_0_-based detection metric and (**b**) *M of N* detection outputs for GPS L1/L2C and L5 frequencies—PRN 10.

**Figure 9:**
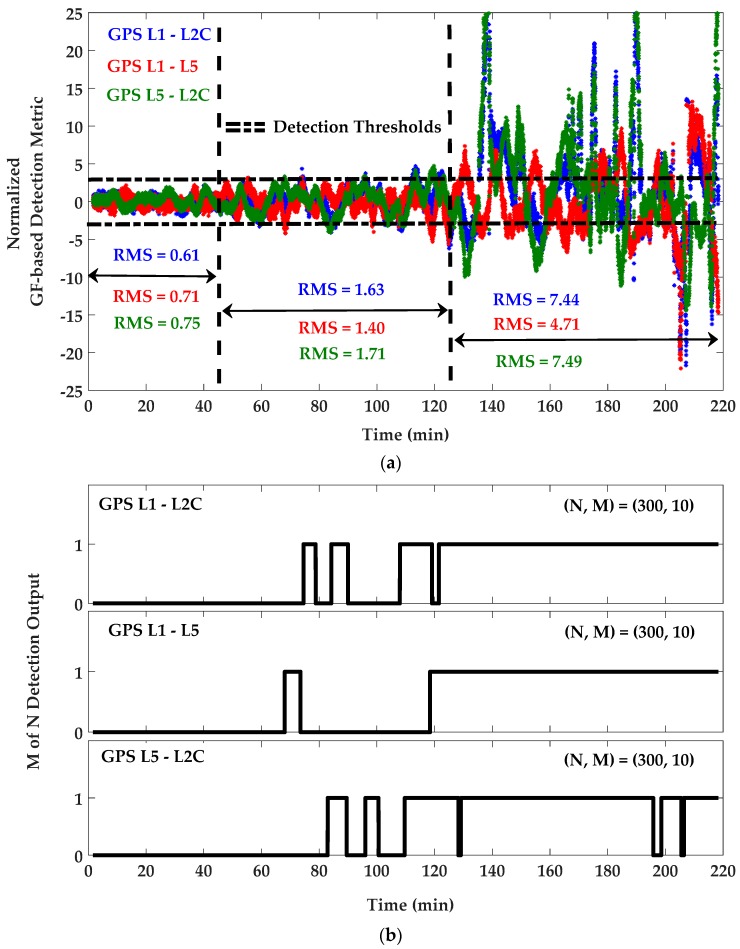
(**a**) GF-based detection metric and (**b**) *M of N* detection outputs for GPS L1/L2C and L5 frequencies—PRN 10.

For detection purposes, C/N_0_ measurements were first filtered to remove their mean values and were then normalized based on an estimation of C/N_0_ SD previously measured by the receiver in an open sky low multipath environment. For the resulting unit-free monitoring metrics, the detection thresholds were set to ±3 as three times the normalized nominal SD for L1, L2C and L5 frequencies. Figure 7 shows the C/N_0_-based monitoring metric and corresponding *M of N* detection results. In this process, *N* and *M* were chosen 300 and 10 (sample of measurements with the input rate of 1 sample/s), respectively, to satisfy the overall probability of false alarm of 1.4 × 10^−8^. The same parameters were used for DC/N_0_ and GF-based detection metrics defined by Equations (12) and (13). Figure 8 and Figure 9 show the DC/N_0_ and GF-based detection metrics and the corresponding results based on the differences between L1, L2C and L5 GPS signals. For GPS L1 positioning, detection results include those provided by the single-frequency C/N_0_-based detection metric on L1 and DC/N_0_ and GF-based detection metrics using the L1-L2C and L1-L5 combinations. For L2C positioning, detection results are related to the single-frequency metric on L2C and the differential metrics using the L1-L2C and L5-L2C combinations. Similarly, for L5 positioning, detection results are considered based on the single-frequency metric on L5 and the differential metrics on L1-L5 and L5-L2C combinations. For each positioning system, a combination of all or some of the related results will be used based on the designed scenario. 

Compared to the single-frequency C/N_0_-based detection metric, the detection sensitivity of the DC/N_0_ and GF-based metrics have slightly increased, especially for the intervals when the multipath signature is magnified due to the differencing effect explained in Section 3.3. There are intervals in which the detection metrics are within the black boundaries but multipath is detected. This is a result of the *M of N* latency discussed in Section 4.1. Given the periodic nature of GNSS multipath, a side effect of the *M of N* latency is reducing the rate of up and down in detection results. In the case of exclusion, the lower change rate in detection outputs was observed to be beneficial to smooth position results, as intentionally considered in this research. Comparing detection results with the corresponding CMC values shown in Figure 5, it is observed that the detection output remains zero for low multipath measurements. When the multipath error exceeds 3 m, it is mostly detected by all the detection metrics. The detection sensitivity can be adjusted by tuning the detection thresholds and of course in a trade-off between detection and false alarm probabilities. This choice depends on the application and multipath environment. Herein, the detection results are used for measurement exclusion or de-weighting, which are in turn limited by geometry. Therefore, the detection sensitivity should be adopted such that relatively significant multipath errors are detected and the position solution improves by excluding or de-weighting the affected measurements. The sensitivity level of 3 m was examined and found as a proper value to sort out multipath affected measurements and benefit from excluding or de-weighting them under the desired PDOP threshold to be discussed in the next subsection.

#### 6.1.2. Position Results

A Least-Squares (LS) solution was used to provide epoch-by-epoch position results. Tropospheric and ionospheric corrections were applied based on Hopfield [40] and Klobuchar [41] models respectively. A constant weight (no weight) solution was compared with the conventional elevation and C/N_0_-based weighting schemes. While the latter methods showed more or less similar results but better than the constant weight scheme, the C/N_0_-based model [20,21,22,23] was chosen as a benchmark to evaluate the performance of detection and exclusion, CMC-based error correction and the combined methods. 

**Figure 10:**
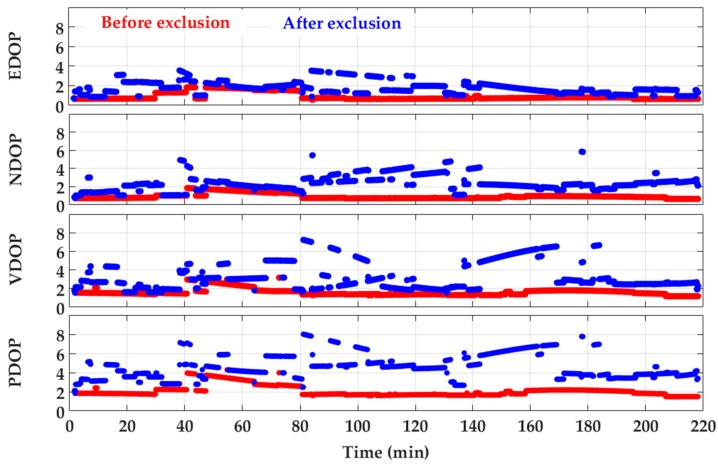
DOP values for GPS L1 positioning before and after exclusion of detected measurements.

First, the performance of the detection and exclusion technique was investigated for GPS L1 positioning. The combination of detection results related to L1 frequency was considered to determine whether a GPS L1 measurement is affected by multipath or not. For exclusion or de-weighting of the detected measurements, the algorithms presented in Section 4.2 were considered. Due to the good visibility of satellites, the exclusion of detected measurements were used to isolate the effect of multipath as it showed higher performance than the iterative de-weighting approach. For exclusion, the consecutive method was used due to its relatively lower complexity compared to subset testing. An empirical value of 8 was chosen as an appropriate PDOP threshold to avoid geometrically poor solutions that would likely not benefit from measurement exclusion. In addition to the PDOP, Figure 10 shows the corresponding east, north and vertical DOP values before and after the detection and exclusion procedure. The DOP values have been calculated based on Equations (6) and (7). At each epoch, the detected measurements were first sorted based on their effect on measurement geometry from low to high and then were consecutively excluded until the tolerable PDOP value was satisfied. As observed, while excluding the affected measurements increases the DOP values, the latter remain under the tolerable thresholds desired to maintain the quality of the measurements geometry at a certain level. This is important since the ultimate position multipath error is a result of both measurement error and geometry.

**Figure 11:**
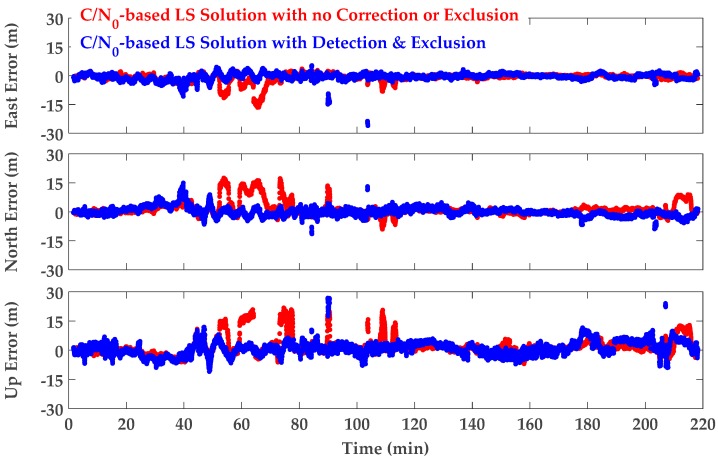
GPS L1 frequency position errors—C/N_0_-based LS solution with no correction or exclusion (red) and after detection and exclusion (blue).

Figure 11 shows position errors for the collected data before (red) and after (blue) detection and exclusion in the local coordinate system. It is observed that there are epochs and intervals where exclusion increases the position errors in all or some directions. However, in conformity with the expectation presented in Section 4.2, by setting the appropriate PDOP threshold, exclusion improves overall position results. In fact, the PDOP threshold value of 8 was empirically determined to benefit from measurement exclusion most of the time. Increasing the threshold (to a larger PDOP) will increase the probability of position degradation (as a result of exclusion). The corresponding mean and RMS values are provided in Table 1 and labeled as A1 and A2. It is observed that positioning performance has improved in all three dimensions. Comparing 3D positioning RMS values, the detection/exclusion technique improves the performance of the C/N_0_-based weight Least-Squares (LS) solution by 27%. 

**Figure 12:**
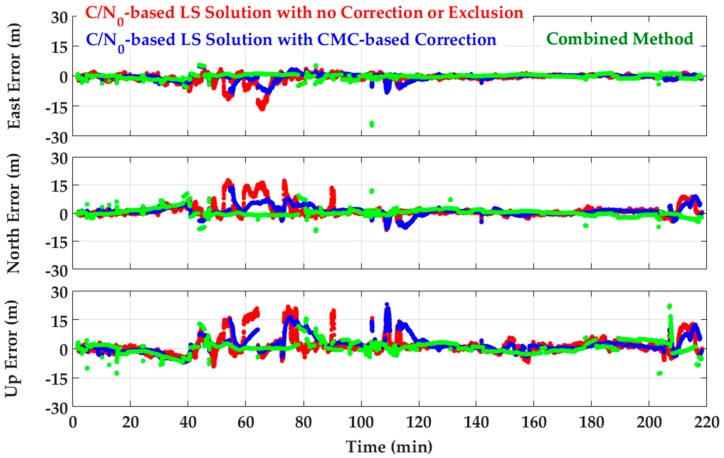
GPS L1 frequency position errors—Red: C/N_0_-based LS solution with no correction or exclusion (benchmark)—Blue: C/N_0_-based LS solution with CMC-based error correction—Green: Combination of a preceding CMC-based error correction followed by the GF-based multipath detection and exclusion in a C/N_0_-based LS solution.

The performance of other monitoring solutions was also compared with the conventional C/N_0_-based LS solution as shown in Figure 12. First a C/N_0_-based LS solution with no exclusion or correction was considered, whose positioning results are in red. 

**Figure 13:**
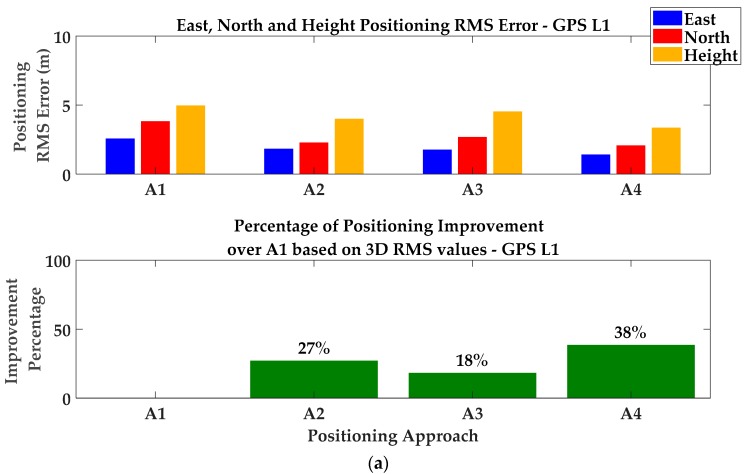

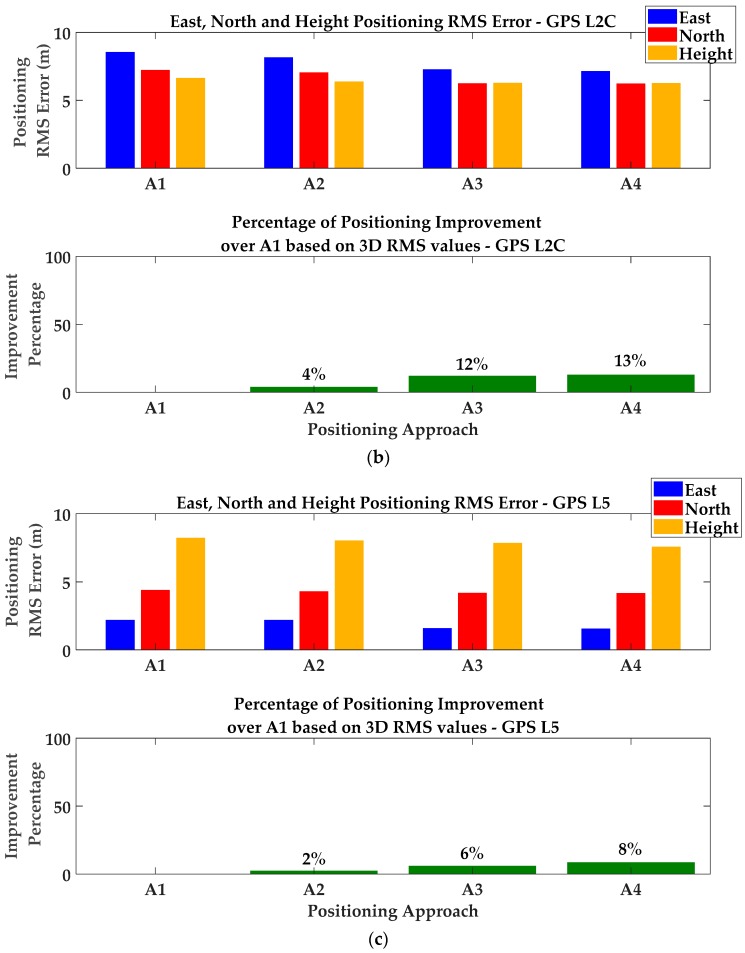
Positioning RMS errors and 3D improvement percentages for GPS (**a**) L1, (**b**) L2C and (**c**) L5 frequencies—Static test scenario.

In the next step, CMC-based multipath corrections were examined where the estimated zero mean CMC values were applied to pseudoranges to alleviate code multipath errors (see Equation (10)). For this scenario, position errors are shown in blue. It was generally observed that CMC-based error corrections effectively smooth position errors except during intervals when cycle-slips degrade the performance of the monitoring approach. Numerical results are presented in Table 1 and Figure 13a (labeled as A3). Based on the corresponding 3D RMS values, the CMC-based error correction shows a 18% improvement over a C/N_0_-based LS solution with no correction. Lower performance is expected as the multipath environment worsens. 

The combination of CMC error corrections and GF detection and exclusion was investigated next. Referring to the discussion in Section 5, due to the good visibility of satellites on different frequencies (mostly on L1 and L2C), the first cascading approach was used. Multipath errors were first alleviated by applying CMC-based error corrections on pseudoranges and then the GF detection metrics were performed on L1–L2C and L1–L5 combinations using the (partially) corrected measurements. Detection results are then exploited to exclude affected measurements in the consecutive exclusion approach discussed in Section 4.2. For included measurements, the C/N_0_-based stochastic model is applied to determine the corresponding variance factors. Results have been plotted in green in Figure 12. Numerical results have been presented in Table 1 and Figure 13a (labeled as A4). It is observed that positioning performance improved by 38% for 3D positioning RMS values which is higher than those resulting from the single detection/exclusion or CMC-based error correction techniques. This confirms the effectiveness of cascading monitoring techniques presented in Section 5, resulting in additional performance for multipath mitigation.

For L2C and L5 positioning, a similar procedure was used (as with the L1 frequency) but based on their corresponding detection metrics. The only major difference was in the L5 case where due to low satellite visibility, the iterative change of measurement weights (de-weighting approach) was used (instead of excluding measurements) to keep all measurements. Referring to Section 4.2, a linear function of iteration number was used to de-weight detected measurements with the maximum number of iterations set to 100 and observed enough to reach the predefined geometry threshold. Table 1 shows the corresponding position errors for L1, L2C and L5 signals. The graphical presentations are shown in Figure 13. The number of satellites broadcasting signals on L2C and L5 frequencies is generally lower than L1, hence geometry is poorer in the former case and position errors are higher; the detection/exclusion or detection/de-weighting techniques therefore yield a lower performance compared to the GPS L1 positioning solution. The mean and RMS values have been extracted for the intervals where the number of satellites is four or more and the position solution has converged. It was also observed that for L2C and L5 positioning (compared to L1), the position results are more biased during several intervals of the data sets due to poorer geometry. While these positive and negative sign biases partly cancel out their effects on the overall mean value, they limit improvement in the RMS values when CMC corrections smooth position results. In general, the lowest RMS values relate to the proposed combined method where multipath errors are first alleviated by applying CMC-based error corrections and then the corresponding GF detection metrics are used to detect and exclude (or de-weight) remaining multipath errors. 

### 6.2. Pedestrian Kinematic Test

**Figure 14:**
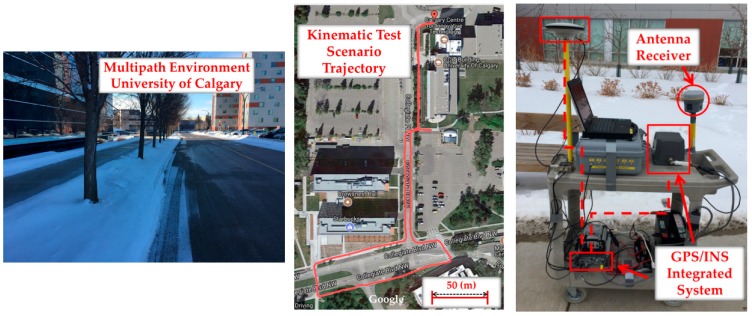
Pedestrian kinematic test scenario—University of Calgary.

Figure 14 shows the data collection setup, trajectory and multipath environment for a pedestrian kinematic test. The same receiver was mounted on a cart moving at a velocity of 0.5 to 2 m/s (to simulate a pedestrian motion) through a suburban area. A reference trajectory was obtained using a NovAtel SPAN (NovAtel Inc., Calgary, AB, Canada) system consisting of a tactical grade Inertial Measurement Unit (IMU) and a GPS receiver. Similar to the static test, the phase velocity trend method [36] was used for cycle slip detection with 2 cycles as the detection threshold. Compared to the static scenario, the length of the moving average was empirically reduced to 60 s to account for the dynamic characteristics of the data that result in faster multipath variations. The *N* and *M* were chosen as 10 and 4 to satisfy the overall false alarm probability equal to 1.1 × 10^−8^. Only GPS L1 was investigated as the mean number of measurements on L2C, and L5 was lower than four limiting positioning outputs to a few epochs. 

**Figure 15:**
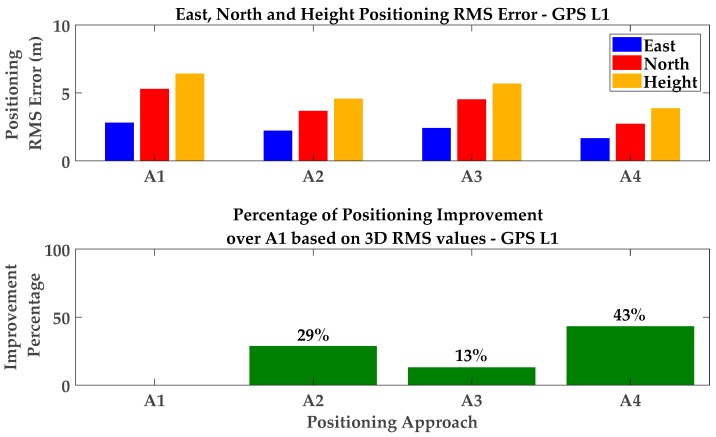
Positioning RMS errors and 3D improvement percentages for GPS L1—Pedestrian test scenario and GPS L1 positioning.

Exclusion of affected measurements showed slightly higher performance compared to the iterative change of measurement weights (de-weighting approach). For exclusion, the consecutive exclusion method was used due to its relatively lower complexity. While more or less similar monitoring results (but with faster multipath variations as a function of time) were observed as those for the static scenario, numerical results are presented here for performance evaluation. Table 2 and Figure 15 show the corresponding results for different positioning approaches. Similar to the static test scenario, a C/N_0_-based LS solution was considered to evaluate the performance of the detection/exclusion technique. The Consecutive exclusion was used and the detected measurements were consecutively excluded based on their impact on measurement geometry. The exclusion continued until the tolerable PDOP threshold of 8 was exceeded. The corresponding mean and RMS values have been presented in the first two rows of Table 2. A graphical presentation of the RMS values has also been provided in Figure 15. It is observed that detection and exclusion of affected measurements improves positioning performance by 29% in 3D positioning RMS values. 

In the next step, the positioning performance was investigated for the CMC-based multipath correction and the combined approach, presented in Section 3.1 and Section 5, respectively. Due to the limited number of simultaneous measurements on all three frequencies (which limited the application of the GF-based detection metrics), the combined method was performed based on first excluding detected measurements using all detection metrics and then correcting the multipath errors on the included PRNs by applying the CMC-based error correction method (Figure 3b). The corresponding numerical results are presented in Table 2 and Figure 15. It is observed that the combination of different monitoring approaches shows a 43% improvement in 3D positioning with respect to the C/N_0_-based LS solution with neither exclusion nor correction. This outperforms the single detection/exclusion and CMC-based error correction techniques through 29% and 13% improvements respectively. 

### 6.3. Land Vehicle Test 

**Figure 16:**
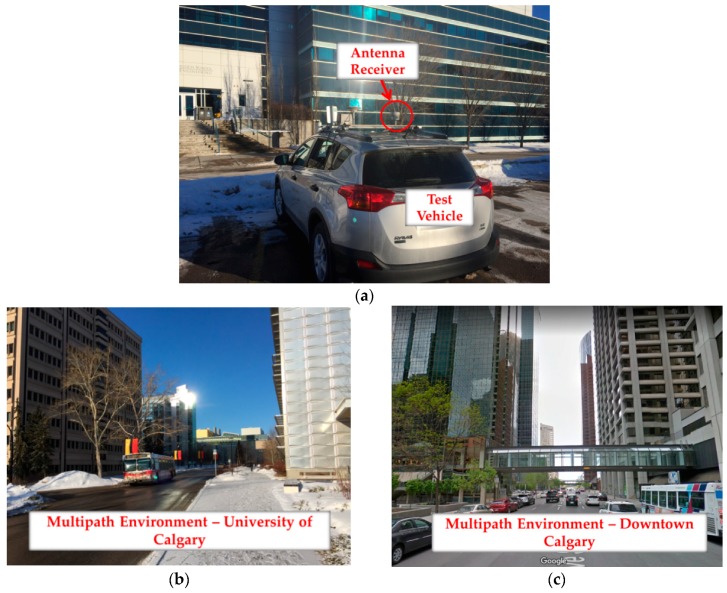
Data collection for land vehicle test—Setup and multipath environments, (**a**) test vehicle (**b**) campus multipath canyon and (**c**) downtown Calgary.

In this third and final test, the same receiver was mounted on a vehicle and data was collected in the different road environments shown in Figure 16. The length of the moving average was empirically reduced to 30 s due to the dynamic characteristics of the data and consequently faster multipath variations. The *N* and *M* were chosen as 5 and 3 to satisfy the theoretical false alarm probability of 0.2 × 10^−8^. As mentioned previously, a cycle slip detection procedure was performed based on the phase velocity trend method and the corresponding threshold was set to 3 cycles due to higher dynamics (as discussed by [36]). Similar to the pedestrian test, only GPS L1 positioning is analyzed where only L1 pseudoranges are used in the position solution, but measurements on other frequencies are used for monitoring purposes. 

The data was categorized into three groups for low-multipath environment, campus multipath canyon (surrounded by buildings with smooth surfaces as shown in Figure 16b) and downtown Calgary (Figure 16c). In the first two data sets, due to good satellite visibility, consecutive exclusion of detected measurements was used to improve the position solution. In the downtown area, due to low visibility, iterative change of measurement weights (de-weighting approach) was used to keep all the measurements, but to iteratively de-weight those affected and reduce multipath errors. Referring to Section 4.2, a linear function was used to de-weight detected measurements and the maximum number of iterations was set to 100, observed enough to reach the predefined geometry threshold. In all cases, the PDOP threshold was again set to 8. Similar to the pedestrian test, the combination of monitoring techniques was performed by first excluding or de-weighting detected measurements (using all detection metrics) and then correcting the multipath errors on the included (or not de-weighted) PRNs by applying the CMC-based error correction. 

**Figure 17:**
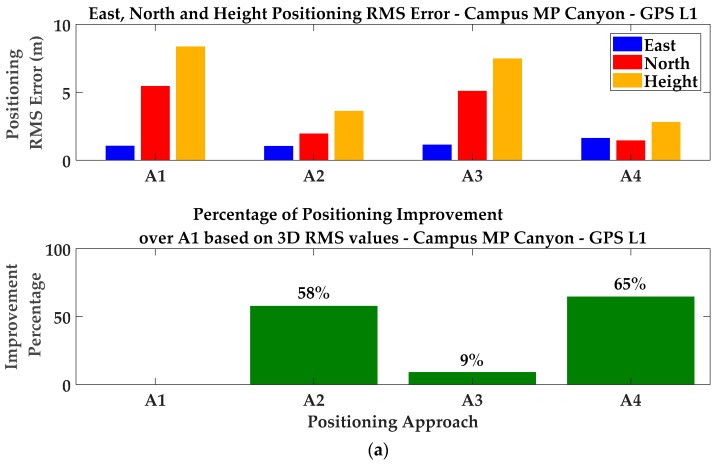

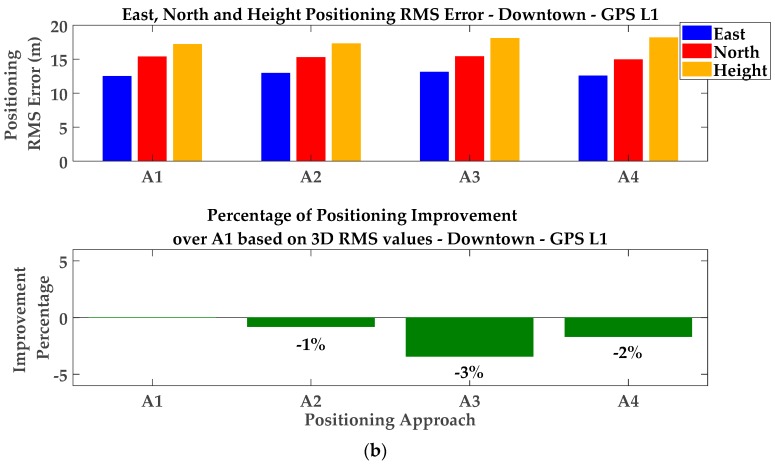
Positioning RMS errors and 3D improvement percentages for (**a**) campus multipath canyon and (**b**) downtown area—GPS L1 positioning.

Position results are presented in Table 3 and Figure 17, they are extracted over the intervals the position solution has converged on. While position results have significantly improved in the campus multipath environment, no improvement is observed in the downtown area. The latter case is because the CMC-based error correction has low performance due to the multiplicity of cycle-slips caused by obstructions and antenna motion in the downtown test. As discussed in Section 3.1, the carrier phase measurements are affected by cycle slips resulting in a new unknown ambiguity. Due to the dependency of the CMC on carrier phase measurements, there is a need to restart the time averaging process in the event of a cycle slip. Therefore, the multiplicity of cycle slips limits the performance of the moving average and thus the correction approach. Moreover, investigation of all data sets revealed that the exclusion or de-weighting of distorted measurements breakdown when more than 50% of measurements are distorted. This was the case in downtown for most of the epochs. It was also observed that a higher dynamic in multipath environments, like the vehicular test scenario in downtown, results in lower performance due to the lag imposed by the time-averaging process. In this case, since multipath behaves more like noise (i.e., stochastic errors) than bias, the C/N_0_-based stochastic model of measurements and weighting scheme shows slightly higher performance before applying exclusion or correction approaches.

Comparing all test scenarios, it is observed that each monitoring technique improves positioning performance (except for the downtown test) and each has its own limitations. The use of only a C/N_0_-based weighting shows generally low performance in mitigating multipath errors. The improvement obtained with the Code-Minus-Carrier (CMC)-based corrections decreases from static to vehicular scenarios with performance mostly limited by cycle slips caused by obstructions and antenna motion. For the detection/exclusion and detection/de-weighting techniques, the performance is limited by measurement geometry and the percentage of affected measurements. For all but the downtown test scenario, the combined methods show higher performance compared to each single technique. As discussed previously, in the downtown test, since the multipath behaves more like noise than bias, the C/N_0_-based weighting shows slightly higher performance before applying any exclusion or correction. 

## 7. Conclusions

Different multipath monitoring techniques were investigated under three major groups as the multipath error estimation and correction, stochastic weighting, and detection and exclusion (or de-weighting) of distorted measurements. While exclusion or de-weighting is challenging due to its effect on geometry, for a dual-frequency receiver, a new Geometry-Free (GF)-based detection metric was investigated given its capability to be combined with a preceding CMC-based error correction to potentially reduce the number of excluded (or de-weighted) measurements in position solutions. Three geometry-based algorithms, namely measurement subset testing, consecutive exclusion and iterative change of measurement weights, were presented to reduce the effect of multiple simultaneous multipath below a tolerable PDOP threshold. Analysis of GPS data showed that while the monitoring techniques can improve position performance under different multipath scenarios, neither technique is 100% effective and each has its own limitations. Hence, the complementary combination of all approaches was investigated in two cascading methods. Comparing 3D positioning RMS values, the proposed combined approaches showed more than 38% improvement over a conventional C/N_0_-weighted LS solution (and also higher than those of the other techniques) for static, pedestrian and vehicular tests. However, the proposed combined method did not show improvement in a city core area with a limited number of satellites in view and higher dynamics.

## Figures and Tables

**Table 1:** Position errors for GPS L1, L2C and L5 positioning and for different positioning approaches—Static test scenario.

Signal	Positioning Approach	East Mean (m)	North Mean (m)	Height Mean (m)	East RMS (m)	North RMS (m)	Height RMS (m)
GPS L1 C/A	A1	−0.95	1.49	1.76	2.56	3.81	4.95
	A2	−0.54	−0.03	0.92	1.81	2.27	3.98
	A3	−0.49	1.07	2.02	1.75	2.67	4.52
	A4	−0.06	0.36	0.02	1.40	2.05	3.34
GPS L2C M + L	A1	−2.56	1.65	1.83	8.54	7.22	6.63
	A2	−1.81	−0.25	2.82	8.15	7.04	6.37
	A3	−2.38	1.56	1.80	7.27	6.24	6.26
	A4	−1.47	−0.09	0.03	7.14	6.22	6.25
GPS L5 I + Q	A1	−0.14	−1.77	4.95	2.18	4.38	8.21
	A2	−0.18	−1.77	4.76	2.18	4.29	8.01
	A3	0.01	−2.27	5.16	1.57	4.18	7.83
	A4	0.03	−1.89	4.86	1.54	4.15	7.55

A1: C/N_0_-based LS solution with no correction, exclusion (or de-weighting); A2: C/N_0_-based LS solution with detection and exclusion (or de-weighting); A3: C/N_0_-based weight LS solution with CMC-based error correction; A4: Combination of a preceding CMC-based error correction followed by the GF-based multipath detection and exclusion (or de-weighting), and a C/N_0_-based weighting.

**Table 2:** Position errors for GPS L1—Pedestrian kinematic test.

Signal	Positioning Approach	East Mean (m)	North Mean (m)	Height Mean (m)	East RMS (m)	North RMS (m)	Height RMS (m)
GPS L1 C/A	A1	−0.55	−1.15	3.49	2.79	5.27	6.40
	A2	−0.94	−0.75	1.75	2.20	3.66	4.56
	A3	−0.38	−0.82	3.06	2.40	4.50	5.67
	A4	−0.69	−0.44	1.21	1.65	2.70	3.85

A1: C/N_0_-based weight LS solution with no correction or exclusion; A2: C/N_0_-based weight LS solution with detection and exclusion; A3: C/N_0_-based weight LS solution with CMC-based error correction; A4: Combination of a preceding multipath detection and exclusion followed by a CMC-based error correction in a C/N_0_-based LS solution.

**Table 3:** Position errors for GPS L1—Land vehicle test.

Signal	Positioning Approach	East Mean (m)	North Mean (m)	Height Mean (m)	East RMS (m)	North RMS (m)	Height RMS (m)
Low Multipath Area	A1	−1.10	−0.05	1.38	1.26	0.49	1.49
	A2	−1.09	−0.13	1.31	1.27	0.52	1.48
	A3	−1.08	−0.03	1.32	1.25	0.46	1.44
	A4	−1.07	−0.11	1.29	1.25	0.49	1.45
Campus MP Canyon	A1	−0.07	1.82	4.49	1.05	5.44	8.35
	A2	0.05	0.42	2.67	1.03	1.95	3.62
	A3	0.62	2.13	3.68	1.14	5.09	7.48
	A4	0.36	0.68	1.73	1.63	1.44	2.80
Downtown Area	A1	−0.11	2.08	3.29	12.50	15.38	17.19
	A2	0.35	2.38	3.58	12.95	15.27	17.29
	A3	−0.15	2.06	3.35	13.12	15.41	18.09
	A4	0.38	2.37	3.57	12.55	14.95	18.19

A1: C/N_0_-based LS solution with no correction, exclusion (or de-weighting); A2: C/N_0_-based LS solution with detection and exclusion (or de-weighting); A3: C/N_0_-based LS solution with CMC-based error correction; A4: Combination of a preceding multipath detection and exclusion (or de-weighting) followed by a CMC-based error correction and a C/N_0_-based weighting.

## Appendix A. Simple and Cumulative Moving Average

In statistical analysis, if *x* is a random process for which a time series of *n* measurements is available, a Simple Moving Average (SMA) of *x* at the *k*th epoch, denoted by ${\langle x\rangle }_{k}$, is the unweighted mean of the previous *n* data:

(A1) $${\langle x\rangle }_{k}=\frac{1}{n}\sum_{l=k-n+1}^{k}x\left(l\right)=\frac{x\left(k\right)+x\left(k-1\right)+\cdots +x\left(k-n+1\right)}{n}.$$

In a Cumulative Moving Average (CMA), the mean value is computed based on the average of all of the data up until the current epoch on a recursive basis:

(A2) $${\langle x\rangle }_{k}=\frac{1}{n}x\left(k\right)+\frac{n-1}{n}{\langle x\rangle }_{k-1}.$$

In Equations (A1) and (A2), if n>k, *n* is replaced by *k* [42].

## Appendix B: CMC-Based Error Correction as Carrier-Smoothing of Code Pseudoranges

The main benefit of the CMC metric can be used for pseudoranges. This is another presentation of carrier-smoothing of pseudoranges. To this end, recall the equation of CMC-based error correction given by Euation (10) and repeated here for convenience. For the *i*th satellite at the *k*th monitoring epoch, one has

(A3) $${\hat{P}}_{i}^{}\left(k\right)=P_{i}^{}\left(k\right)-\left(CMC_{i}^{}\left(k\right)-{\langle CMC_{i}^{}\rangle }_{k}\right),$$

where ${\hat{P}}_{i}^{}\left(k\right)$ and $P_{i}^{}\left(k\right)$ are respectively corrected (or estimated) and measured pseudoranges for the *i*th PRN at the *k*th monitoring epoch. $CMC_{i}^{}\left(k\right)$ is the corresponding CMC value and ${\langle CMC_{i}^{}\rangle }_{k}$ denotes the mean value of the CMC metric at the *k*th epoch computed using a moving average. By replacing the definition of the CMC metric in Equation (A3), one obtains

(A4) $$\begin{array}{cc} {\hat{P}}_{i}^{}\left(k\right) & =P_{i}^{}\left(k\right)-\left(P_{i}^{}\left(k\right)-\phi_{i}^{}\left(k\right)-{\langle P_{i}^{}-\phi_{i}^{}\rangle }_{k}\right)\\ & =\phi_{i}^{}\left(k\right)+{\langle P_{i}^{}-\phi_{i}^{}\rangle }_{k}, \end{array}$$

where $\phi_{i}^{}\left(k\right)$ is the corresponding carrier phase measurement (in metres) for the *i*th PRN at the *k*th monitoring epoch. Assuming a cumulative moving average (see Appendix A), Equation (A4) can be rewritten as

(A5) $$\begin{array}{cc} {\hat{P}}_{i}^{}\left(k\right) & =\phi_{i}^{}\left(k\right)+\frac{n-1}{n}{\langle P_{i}^{}-\phi_{i}^{}\rangle }_{k-1}+\frac{1}{n}\left(P_{i}^{}\left(k\right)-\phi_{i}^{}\left(k\right)\right)\\ & =\phi_{i}^{}\left(k\right)+\frac{n-1}{n}\left({\hat{P}}_{i}^{}\left(k-1\right)-\phi_{i}^{}\left(k-1\right)\right)+\frac{1}{n}\left(P_{i}^{}\left(k\right)-\phi_{i}^{}\left(k\right)\right). \end{array}$$

Exploiting some basic mathematical calculations, Equation (A5) can be rewritten as

(A6) $${\hat{P}}_{i}^{}\left(k\right)=\frac{1}{n}P_{i}^{}\left(k\right)+\frac{n-1}{n}\left({\hat{P}}_{i}^{}\left(k-1\right)+\phi_{i}^{}\left(k\right)-\phi_{i}^{}\left(k-1\right)\right),$$

which is a definition of carrier-smoothing of code pseudorange [1].
